# Supplementary figures and images for: Development and validation of ferroptosis-related lncRNAs signature for hepatocellular carcinoma
Source: PeerJ. 2021 Jun 11;9:e11627. doi: 10.7717/peerj.11627 (PMC8202323; doi:10.7717/peerj.11627)

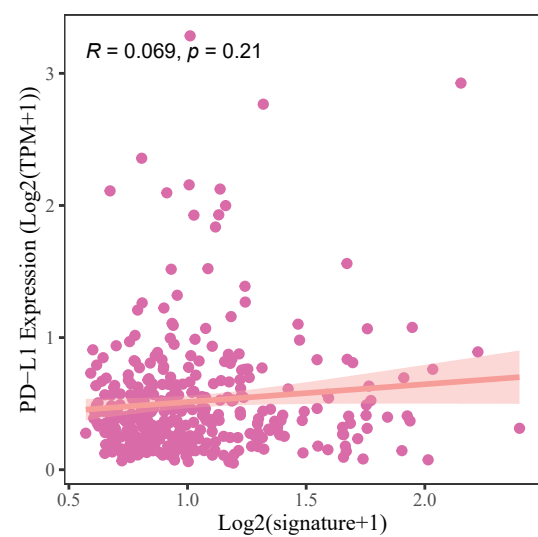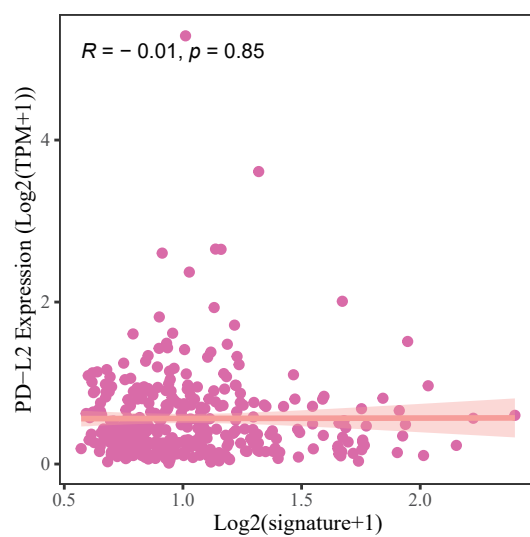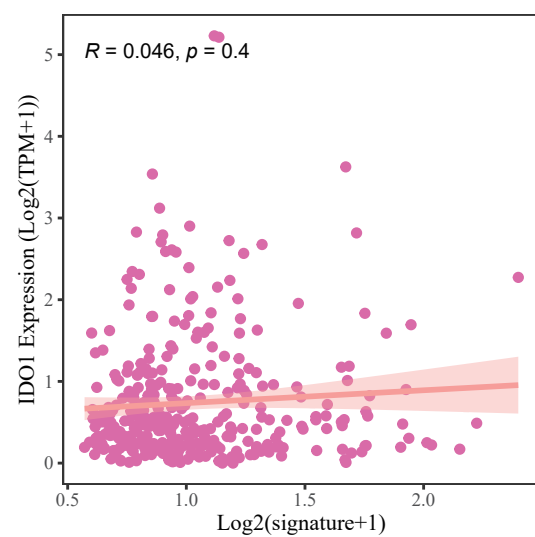

Supplement: Supplemental Information 1 [file peerj-09-11627-s001.pdf]
